# Primary adrenal malignant melanoma: A case report and review of literature

**DOI:** 10.1097/MD.0000000000008956

**Published:** 2017-12-22

**Authors:** Bo Xu, Yazhao Hong, Meishan Jin, Mingyang Li, Chunxi Wang, Xiaoqing Wang

**Affiliations:** a Department of Urology; b Department of Pathology, The First Hospital of Jilin University, Changchun Jilin, P.R. China.

**Keywords:** adrenal gland, ipilimumab, melanoma, primary, treatment

## Abstract

**Rationale:**

The primary adrenal melanoma (PAM) was an extremely rare occurrence, which was demonstrated as the few cases described in the medical literature.

**Patient concerns:**

We reported a 58-year-old man who was admitted to hospital because of intermittent left flank pain which lasted for a month. The renal computed tomography (CT) scan showed that a large retroperitoneal tumor measuring 15.5 cm × 12.1 cm × 13.0 cm seemed to have its origin in the left adrenal gland.

**Diagnoses:**

According to clinical symptoms, previous history, physical examination, and postoperative pathology, the patient was diagnosed as PAM.

**Interventions:**

The patient was treated with an open procedure for resection of retroperitoneal tumor. After the surgery, the patient participated in the clinical drug trial and received treatment with ipilimumab as adjuvant medical therapy.

**Outcomes:**

When this article was completed, the patient was still alive and the survival has been already up to 20 months.

**Lessons:**

The PAM was extremely rare in clinic, and its diagnosis and differential diagnosis were difficult. Therefore, clinical physicians should attach great importance to this disease.

## 1 Introduction

Primary adrenal melanoma (PAM) is a clinically extremely rare malignant tumor, which was demonstrated as only a few cases reported.^[1–13]^ Most of the cases were accidental discovery. The characteristics of clinical symptoms and signs are not obvious, and the most common symptom is flank pain, and it is impossible to obtain typical features from imaging examination. Thereby, the diagnosis and differential diagnosis of PAM mainly are based on histological and immunohistochemical studies. Here, we reported an unusual case of PAM with pulmonary metastases and summarized the characteristics of PAM to improve the understanding of PAM.

## 2 Case report

**Figure 1:**
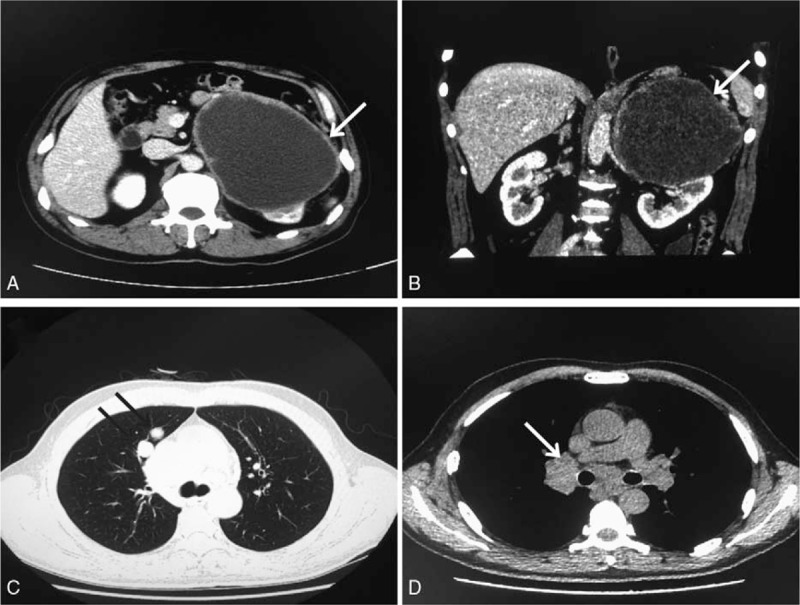
(A, B) Renal plain CT and enhancement CT scan showed a tumor in the upper region of the perinephric space. (C, D) Pulmonary CT scanned right upper lobe metastases and enlarged lymph nodes of mediastinum. CT = computed tomography.

The study was approved by the Ethics Committee of the First Hospital of Jilin University. The patient signed an informed consent document allowing his clinical history and photographs to be published. A 58-year-old man was admitted to our hospital because of intermittent pain in left flank in the past month. Enhancement CT scan showed that a circular mass (15.5 cm × 12.1 cm ×  3.0 cm) above left kidney was considered as malignancy and the left adrenal gland was not found (Fig. 1A, B). Pulmonary CT scan revealed that right upper lobe metastases and enlarged lymph nodes of mediastinum and hilum of right lung were considered as malignancy (Fig. 1C, D). The patient did not have history of hypertension and the hormone examinations were normal. Based on the clinical symptoms and signs, and also the CT findings, the patient was diagnosed with retroperitoneal tumor. It seemed to have its origin in the left adrenal gland which was surrounded by the tumor, although the possibility of another type of primary retroperitoneal tumor could not be excluded. An open procedure for resection of retroperitoneal tumor was performed. The tumor which constricted the kidney with displacement and deformity was sharply demarcated with the upper pole and the renal blood vessels of the left kidney. The tumor was completely removed. As a consequence, the patient recovered without any postoperative complications.

**Figure 2:**
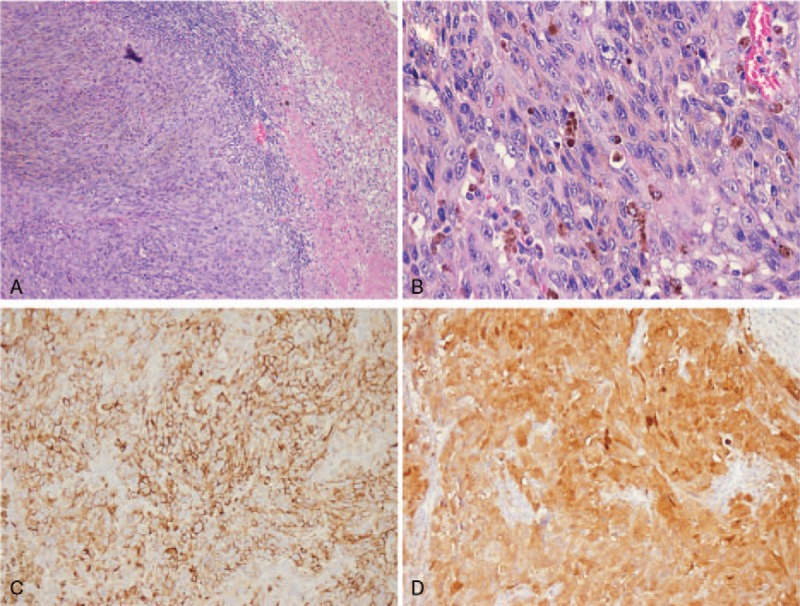
(A) The tumor was characteristic of sheet arranged cells and infiltrative growth (HE ×100). (B)The tumor cells were different in size and rich in cytoplasm, a few cells were cytoplasm transparent. A few melanin granules were scattered (HE ×400). (C, D) Immunohistochemical staining of HMB-45 and S-100 showed intense and diffuse positivity (MaxVision method ×200). HE = hemotoxylin and eosin, HMB-45 = human melanoma black-45.

The anatomopathological study revealed a cystic tumor measuring 13.5 cm × 9 cm × 4 cm, with a wall thickness of about 0.1 to 2.0 cm. The inner wall was reddish brown, blood coagulation and rough, and the section was reddish brown, solid, and blood coagulation. The microscopic study also showed a tumor with ephithelioid cells, with heterotypic cells, with nuclear groove, folding nucleus, intranuclear inclusions, and frequent multinucleation (Fig. 2A, B). On the periphery of the tumor, remnants of the adrenal gland were recognizable, without tumor cells that infiltrated vessels, nerves, or surgical margin. Immunohistochemical staining was negative for inhibin, synaptophisyn, cytokeratin-pan, and chromogranin A. Vimentin and melanocytic markers (S-100, human melanoma black-45) were strongly positive (Fig. 2C, D).

**Figure 3:**
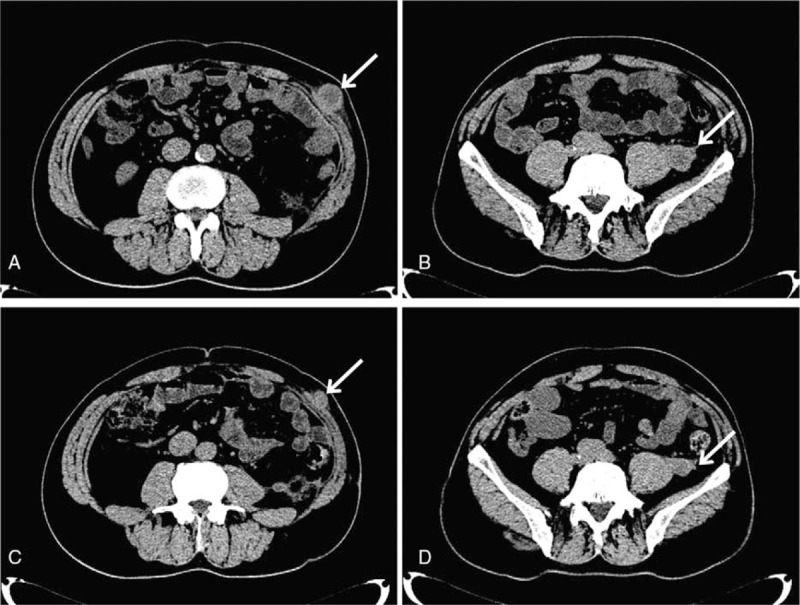
Abdominal CT scanned the left solid abdominal subcutaneous mass and local multiple nodules of the left lateral psoas muscle and the left abdominal wall in 5 months (A, B) and 12 months (C, D) after surgery, respectively. CT = computed tomography.

**Figure 4:**
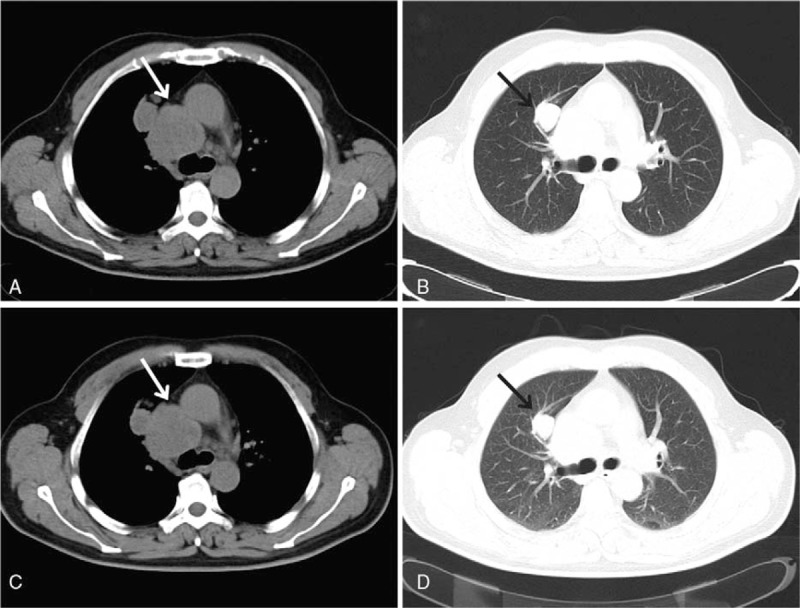
Pulmonary CT scanned that the right upper lobe nodules and mediastinal lymph nodes in 5 months (A, B) and 12 months (C, D) after surgery, respectively. CT = computed tomography.

We did not find any signs which revealed that melanoma existed in other parts of his body before operation, and the patient did not have the history of stained skin resection. Melanoma was not found through further medical examination after surgery, including gastrointestinal endoscopy and fundoscopic eye examination. Postoperative gene mutation analysis was a wild type. The patient did not receive any adjuvant therapy after this surgery. An abdominal CT scan showed that there were multiple masses measuring 1.9–3.2 cm on the left abdominal subcutaneous tissues and left side of posterior peritoneum 5 months after the surgery (Fig. 3A, B). The patient participated in the clinical drug trial and received treatment with ipilimumab as adjuvant medical therapy. Ipilimumab was administered via a 90-minute intravenous infusion at 3 mg/kg on the first, forth, seventh, and tenth weeks. Through the treatment of 12 months, we observed that multiple nodules in left abdominal subcutaneous and left posterior peritoneum measuring 1.6–2.6 cm were smaller than before (Fig. 3C, D). Pulmonary CT showed that the right upper lobe nodules and mediastinal lymph nodes were slightly smaller than before (Fig. 4). Mild fatigue and nausea were the major side effects after ipilimumab treatment.

## 3 Discussion

Primary melanoma is a highly malignant tumor, which is found on the skin frequently, followed by the eyes (choroid plexus), the mouth cavity, the esophagus, the larynx, the bronchi, the rectum, the genitourinary system, the meninges, the adrenal glands, and so on. Most of the adrenal malignant melanomas are metastatic tumors, and the primary tumor is very rare. In 1946, the first observation of PAM was reported by Kniseley and Baggenstoss,^[1]^ but it was not fully consistent with the subsequent diagnostic criteria. In 1976 and 1984, Ainsworth et al^[14]^ and Carstens et al^[4]^ separately established a series of diagnostic criteria for PAM according to clinical, histological, and immunohistochemical studies. At present, most reports use the diagnostic criteria of Carstens et al.^[4]^ These criteria are as follows: only 1 adrenal gland involved; a careful examination to eliminate melanoma in other parts of the patient's body; no surgery history of pigmented mucous, cutaneous, or ocular lesions; ruling out any hidden pigmented lesions, preferably by autopsy results. Subsequently, a great many scholars had reported the cases.^[5–13]^ From 1946 to 2012, 13 cases of patients suffering from PAM were retrieved in MEDLINE. There are a total of 14 patients of PAM including our case.

Primary adrenal melanoma is usually a larger tumor with the largest diameter range from 8 to 17 cm,^[9]^ and only invades the unilateral adrenal gland. Middle-aged and old people are more susceptible to it. Most of them are nonfunctional tumors. The original occurrence of PAM is still not clear. Related studies suggested that it might be due to pluripotent neural crest cells,^[8,15]^ which could migrate after induction and enter into the multicell lineage including melanocytes, neurons, glial cells of the peripheral nervous system, and adrenal chromaffin cells through cell differentiation. Melanocytes could be located in the adrenal gland, and cause metaplasia and malignant transformation, leading to the formation of melanoma.^[8,15,16]^ This would explain the reason why primary melanoma could originate in the adrenal glands. PAM has no obvious clinical symptoms and signs, and pain is the most common clinical manifestation. There is also a report of the PAM patient with imprecise gastrointestinal disorders, caused by tumor compression of adjacent gastrointestinal system. Typical clinical manifestations of malignant tumors—fatigue, loss of appetite, and weight loss—are less likely to occur in the PAM patients. The CT usually scans the tumor with contrast enhancement. Common metastases in PAM patients are lung, liver, gastrointestinal tract, brain, and bone, and our patient was considered as having lung metastasis.

The main difficulty of clinicians in the differential diagnosis of PAM is how to distinguish PAM from metastatic tumors. At the same time, the differential diagnosis is very important, because the treatments are drastically different. Since there is no significant difference between PAM and metastatic tumors based on the results of CT, magnetic resonance imaging, and pathology, the identification of PAM is mainly dependent on the criteria of Carstens et al. Primary lesions could be found in patients with adrenal metastases of malignant melanoma. The characteristic of the CT scanning of adrenal metastases is largely adrenal heterogeneous lesions with central or irregular areas of necrosis/haemorrhage and a thick contrast-enhancing rim.^[17]^ Bilateral metastasis with characteristic appearance is a kind of adrenal metastasis.^[18]^ It may be also misdiagnosed as PAM because of the hidden melanoma of the skin or other parts, and surgical history of pigmented mucosa, skin, or ocular lesions. Pigmented pheochromocytoma, pigmented adrenal adenoma, adrenal hematoma, and adrenocortical carcinoma also have to be considered in the differential diagnosis which mainly depends on immunohistochemistry and electron microscopy.

Primary adrenal melanoma is usually treated by surgical resection of intact tumors, the ipsilateral adrenal gland, and kidney.^[12]^ However, surgical treatment of PAM is not extremely effective, with a mortality rate approaching 100% within 2 years.^[6]^ The longest survival (period) reported is only 46 months.^[7]^ There has not been a consensus on postoperative adjuvant therapy of PAM because of the infrequency. At present, adjuvant therapy for malignant melanoma mainly includes targeted therapy and immunotherapy. The tumor with genetic mutation is often treated with targeted therapy, such as vemurafenib and dabrafenib.^[19,20]^ Immunotherapy for malignant melanoma mainly includes antihuman cytotoxic T-lymphocyte antigen-4 (CTLA4) monoclonal antibody, programmed death 1 (PD-1) inhibitor, and programmed death-ligand 1 (PD-L1) inhibitor.^[21]^ All of them are immune checkpoint inhibitors acting through receptor blockade at specific points in the immune response. In 2011, ipilimumab, a kind of anti-CTLA4 monoclonal antibody, was approved by the US Food and Drug Administration for the first-line clinical treatment of advanced melanoma.^[22]^ PD-1, a T-lymphocyte surface receptor, suppresses cell immune response through interaction with ligands including PD-L1 and PD-L2.^[21,23]^ PD-1 inhibitor (lambrolizumab and nivolumab) and PD-L1 inhibitor (MPDL3280A) enhance antitumor immunity through the blockade of PD-1 interaction with its ligands.^[21,24]^ Since there were solely phase I/II clinical trials, their toxicity or resistance has not been clear and defined. CTLA4, a co-inhibitory molecule, leads to an inhibitory signal of the T-cell through expression on the T-cell surface after being activated. By means of CTLA4 blockade, ipilimumab suppresses the inhibitory signal of the T cell, thereby enhancing immune response. Related research reported that ipilimumab could lead to some side effects which vary from immune-related adverse events (pruritus, rash, diarrhea, etc) to nonspecific symptoms (cough, headache, fatigue, etc) as a result of the lack of antigen specificity.^[25]^ In addition, many clinical studies have shown that ipilimumab could significantly improve thousands of patients’ survival.^[26–28]^ In our case, ipilimumab was also used for the treatment of postoperative adjuvant immunotherapy. And then, the abdominal and pulmonary CT scanned that most of the metastatic tumors were smaller than those 12 months ago. When this article was completed, the patient was still alive and the survival has been already up to 20 months.

## 4 Conclusions

The clinical case which we reported met the criteria of Carstens et al. The melanoma which was not found in the organ of eyes, digestive system, and urogenital system only involved 1 adrenal gland. There was no history of melanoma or pigmented lesion. There were no autopsy results, for our patient was still alive. Pulmonary CT suggested the presence of lung metastases. In the histology and immunohistochemistry, the tumor was consistent with the characteristics of melanoma. We come to the conclusion that our patient's lesion is a PAM.
